# Supplementary material for: Description and validation of the Postoperative Discharge Recovery State outcome: a patient-partnered population-based cohort study
Source: Br J Anaesth. 2025 Jun 25;135(3):746–54. doi: 10.1016/j.bja.2025.05.020 (PMC12489357; doi:10.1016/j.bja.2025.05.020)
Supplement: Multimedia component 1 [file mmc1.docx]

**Appendix 1 – Data sets linked to create analytic dataset**

All data were linked deterministically across the following datasets using an anonymized, unique identifier: the Discharge Abstract Database (**DAD;** hospital admissions, diagnoses and procedures); the Registered Persons Database (**RPDB;** death dates and sociodemographic information); the National Ambulatory Care Reporting System (**NACRS;**  all emergency department visits); the National Rehabilitation Reporting System (**NRS;** admissions and discharges to adult inpatient rehabilitation facilities and programs); the Ontario Health Insurance Claims Database (**OHIP;** fee for service physician claims); the Continuing Care Reporting System (**CCRS;**  clinical and demographic information on residents receiving facility-based continuing care).

**Appendix 2 – Description of patient engagement in creating the Postoperative Discharge Recovery State outcome**

Authors (EH, DK, DIM) developed and piloted a questionnaire with patient partner and co-author, GK. The lead patient partner Gurlavine Kidd (GK) is an established member of the research program who partnered in this study and facilitated the snowball sampling technique to engage 5 additional patient partners (see partner characteristics in table below). Of note, partner ages ranged from 65 to 85, each partner had experience as both a patient and as a caregiver around surgical care, and partners came from rural and urban areas and included both individuals identifying as men and as women.

**Patient Partner Characteristics**

| **Ages** | 65, 67, 67, 73, 74, 85 |
| --- | --- |
| **Gender, n (%)** | Man – 2 (33) |
|  | Woman – 4 (67) |
| **Ethnicity & Race, n (%)** | White – 6 (100) |
| **Geography, n (%)** | Rural - 3 (50) |
|  | Urban - 3 (50) |
| **Education, n (%)** | High school - 1 (14) |
|  | Bachelor’s degree - 3 (29) |
|  | Graduate degree - 2 (57) |
| **Surgical patient** | Yes – 6 (100) |
| **Caregiver to someone who has had surgery** | Yes – 6 (100) |

A member of the research team (EH) connected with each patient partner to explain the purpose of the questionnaire and how their responses would be interpreted and used to support the research study. The questionnaire was administered to each patient partner either by phone (n=4) or email (n=2) based on their preference. Open-ended questions were analyzed using content analysis and the study team (including Patient Partner GK, who is an older adult with lived experience of having major inpatient elective surgery) met to review and interpret the findings. Collectively, key points were identified that informed the Postoperative Recovery Discharge State outcome.

Below is the Patient Partner Questionnaire with some example responses to the open-ended questions.

**Patient Partner Questionnaire**

*Our research team wants to explore what factors (for example: health status, surgery type, age, living status) predict who is more likely to go home after surgery and who is more likely to go to a new location other than home after surgery (for example, long-term care or retirement home). The goal of this questionnaire is to learn what approach to communicating your personal risk of not going home after surgery would be most meaningful to you should you ever have surgery. Your responses will help our research team know where to focus our research so that it matters to patients just like you.*

Specifically, we want to understand the following:

If you were making the decision to have surgery, at what time frame would you most value knowing your chances of being back home?

1 week

1 month

3 months

6 months

1 year

It doesn’t matter how long, as long as I get there

Other 

Please tell me more about why your above answer is the most important time to you.

How long is too long?

What would be ideal and why?

*To me, 3 months is doable. It seems “life-sized” and is doable.*

*I would certainly have to do a lot of work to prepare for the surgery and aftermath with a three month window, but at least if I knew it was definitely not longer than 3 months and the surgery was vital, and my recovery from whatever the problem was a sure thing, I would likely be willing to go for it.*

Other thoughts or perspectives?

*The next most important option would be “It doesn’t matter how long, as long as I get there”. This is important to be because I just want to get home!*

 If you were making the decision to have surgery, what type of information would you value most about the chances of you getting home as compared to needing to go live somewhere else to get the care you need after surgery? Please review the options below select what information you would value most.

Options:

The risk that I would not be at home at the time point highlighted above after my surgery

The average time that someone like me would expect to recuperate before making it home after surgery, along with the risk that I would not make it home within that period of time

The risk that someone like me would die, end up still being in hospital, being alive but not in hospital or at home (e.g., nursing home), or being home

Other?

*I would definitely want to know what the chances were of dying or being incapacitated by the operation. This is most important to me because if the risks of dying or more particularly, ending up in a nursing home, totally incapacitated were high, I might opt not to have the surgery.*

*I would value Option B most. To me, this feels soft and safe and tells me that the clinicians have done this many times and that they have an idea of what I might experience or can expect. This type of average and personalized risk seems like a trustworthy phrase to me. I also wonder how the docs are going to be able to communicate this to me – do they have time? How will they communicate it clearly? They are the ones to know the risks but would I get connected to a social worker to help me understand what this means for me? I do like the idea of a visual to help me understand (i.e. stick people). The next option I would value most would be Option C – specifically, I do not want to go to LTC but would be okay with rehab before going home. But ultimately, I just want to get home. I would want to know about discharge location because it affects my lifestyle and what I need to do to prepare.*

Please tell me more about why your above answer is most important to you? What other information would you want to know?

*I would want to know the average time for recovery for someone my age and any in-home supports available to me. I would want to know what life would look like after surgery. I live alone so I would need to know if I have to stay on my main floor, etc. I have actually decided against having my knee replacement surgeries because nobody could provide me with this information.*

*I would definitely want to know what the chances were of dying or being incapacitated by the operation.*

*Will there be other costs (i.e. for rehab and LTC)? What is it? Will I need home care? How do I get it? Is there a cost? Is there a waiting list? Can my family talk to someone else here if it happens that I can’t go home? I’m so naïve as a patient, I think I am going to be fine if I am going home but will I be? Will I experience any decline? What will it be like being home?*

Aside from dying what would be the worst possible outcome after surgery for you?

*The worst possible outcome would be being totally incapacitated, living for the rest of my life in a nursing home, not being comfortable and not being able to be independent and living at home. If that was the outcome, I would actually prefer dying.*

*The worst would be to be disabled and not able to go home or look after myself.*

*That I was less able than I was before surgery because that’s the only reason I would have surgery. I want to be able to comfortably go for a walk. I’m terrified of falling because I’m terrified I can’t get up. If I had same or worse level of ability that would be devastating. I have friends who said they would not haven chosen to have surgery if they knew this is what they would experience.*

What about the recovering from surgery scares you or worries you the most?

*What worries me the most about recovery after surgery is … having to go LTC and not going home … not living independently and being at the mercy of others who I do not know.*

*Managing it - coming home would of course would be nice but I live alone so could I go home?*

**Appendix 3 – List of included surgeries, corresponding Canadian Classification of Intervention (CCI) codes and Operative Stress Score (OSS) ratings**

| **Description** | **CCI** | **OSS** |
| --- | --- | --- |
| Repair, abdominal aorta | 1KA80 | 5 |
| Excision partial, abdominal aorta | 1KA87 | 5 |
| Dilation, abdominal arteries NEC | 1KE50 | 5 |
| Occlusion, abdominal arteries NEC | 1KE51 | 5 |
| Excision partial, esophagus | 1NA87 | 5 |
| Excision partial with reconstruction, esophagus | 1NA88 | 5 |
| Excision total, esophagus | 1NA89 | 5 |
| Excision total with reconstruction, esophagus | 1NA90 | 5 |
| Excision radical, esophagus | 1NA91 | 5 |
| Excision radical with reconstruction, esophagus | 1NA92 | 5 |
| Excision partial, pancreas with duodenum | 1OK87 | 5 |
| Excision total, pancreas with duodenum | 1OK89 | 5 |
| Excision radical, pancreas with duodenum | 1OK91 | 5 |
| Excision total, lobe of lung | 1GR89 | 4 |
| Excision radical, lobe of lung | 1GR91 | 4 |
| Excision radical, lung NEC | 1GT91 | 4 |
| Bypass, abdominal aorta | 1KA76 | 4 |
| Excision total, stomach | 1NF89 | 4 |
| Excision total with reconstruction, stomach | 1NF90 | 4 |
| Excision radical, stomach | 1NF91 | 4 |
| Excision radical with reconstruction, stomach | 1NF92 | 4 |
| Excision partial, rectum | 1NQ87 | 4 |
| Excision total, rectum | 1NQ89 | 4 |
| Excision partial, liver | 1OA87 | 4 |
| Excision total, bladder NEC | 1PM89 | 4 |
| Excision total with reconstruction, bladder NEC | 1PM90 | 4 |
| Excision radical, bladder NEC | 1PM91 | 4 |
| Excision radical with reconstruction, bladder NEC | 1PM92 | 4 |
| Excision partial, lung NEC | 1GT87 | 3 |
| Excision total, lung NEC | 1GT89 | 3 |
| Extraction, carotid artery | 1JE57 | 3 |
| Bypass, arteries of arm NEC | 1JM76 | 3 |
| Bypass, arteries of leg NEC | 1KG76 | 3 |
| Bypass, vessels of the pelvis, perineum and gluteal region | 1KT76 | 3 |
| Excision partial, stomach | 1NF87 | 3 |
| Excision partial, large intestine | 1NM87 | 3 |
| Excision total, large intestine | 1NM89 | 3 |
| Excision radical, large intestine | 1NM91 | 3 |
| Excision total, kidney | 1PC89 | 3 |
| Excision radical, kidney | 1PC91 | 3 |
| Repair, arteries of leg NEC | 1KG80 | 2 |
| Excision partial, kidney | 1PC87 | 2 |
| NEC: Not elsewhere classified |  |  |

**Appendix 4 – Technical description of operationalizing the Postoperative Discharge Recovery State outcome in routinely collected health data**

Ordinal categorization of possible Postoperative Discharge Recovery States:

-1-Death: death listed as the discharge disposition in the index DAD hospitalization record if discharge occurred before 90-days, or date of death in provincial vital statistics (RPDB) up to 90-days after surgery.

-2-Alive in hospital: discharge date from the index DAD hospitalization record >90 days from surgery, or a new DAD hospitalization record inclusive of postoperative day 90.

-3-Alive in a long-term care facility: An admission in the CCRS overlapping postoperative day 90.

-4-Alive in a rehabilitation hospital: an admission in the NRS overlapping postoperative day 90.

-5-Alive at home: patient is alive (no death date in RPDB) and not admitted to a long-term care or rehabilitation center on postoperative day 90.

**Appendix 5 – Sample size and power considerations**

As a population-based study we included all eligible members of the Ontario population. However, we did estimate the expected power to support regression modelling. While sample size estimates for a multivariable ordinal regression model were not well-defined, methods described for a multinomial logistic regression model suggested that a model with up to 30 parameters and a Cox-Snell R^2^ as low as 0.01 would be stable with 26,064 participants (which we expected to exceed). As experience with these data suggested that no variable would be missing with >1% incidence, we planned for, and proceeded with, a complete case analysis.

**Appendix 6 – Cohort Demographics with Operative Stress Score and Surgical Speciality presented as row percentages**

|  | n=2,718 | n=1,696 | n=179 | n=593 | n=79,236 |  |
| --- | --- | --- | --- | --- | --- | --- |
| Age* | 77.3 (6.9) | 75.2 (6.3) | 80.8 (6.6) | 77.6 (6.6) | 74.6 (6.2) | <0.001 |
| Female sex | 973 (35.8%) | 640 (37.7%) | 105 (58.7%) | 235 (39.6%) | 32,248 (40.7%) | <0.001 |
| Male sex | 1,745 (64,2%) | 1,056 (62,3%) | 74 (41.3%) | 358 (60.4%) | 46,988 (59.3%) | <0.001 |
| Rural residence | 410 (3.2%) | 272 (2.1%) | 31 (0.2%) | 56 (0.4%) | 12,221 (94.1%) | 0.002 |
| Frailty index score* | 0.19 (0.08) | 0.18 (0.08) | 0.22 (0.10) | 0.20 (0.08) | 0.14 (0.07) | <0.001 |
| Charlson Index** | 1 (1-2) | 1 (0-2) | 1 (1-2) | 1 (1-2) | 1 (0-1) | <0.001 |
| Operative Stress Score |  |  |  |  |  | <0.001 |
| *2* | 42 (1.8%) | 30 (1.3%) | 7 (0.3%) | 16 (0.7%) | 2199 (95.9%) |  |
| *3* | 773 (2.3%) | 522 (1.5%) | 66 (0.2%) | 203 (0.6%) | 32119 (95.4%) |  |
| *4* | 1498 (4.1%) | 878 (2.4%) | 91 (0.3%) | 287 (0.8%) | 33625 (92.4%) |  |
| *5* | 405 (3.4%) | 266 (2.2%) | 15 (0.1%) | 87 (0.7%) | 11293 (93.6%) |  |
| Surgical Specialty |  |  |  |  |  | <0.001 |
| *General* | 1486 (3.5%) | 850 (2.0%) | 107 (0.3%) | 268 (0.6%) | 40033 (93.7%) |  |
| *Vascular* | 493 (2.6%) | 348 (1.8%) | 48 (0.3%) | 173 (0.9%) | 18000 (94.4%) |  |
| *Thoracic* | 280 (3.4%) | 206 (2.5%) | NR | NR | 7819 (94.1%) |  |
| *Urology* | 379 (3.2%) | 241 (2.1%) | 13 (0.1%) | 83 (0.7%) | 11014 (93.9%) |  |
| *Other* | 80 (3.2%) | 51 (2.0%) | 8 (0.3%) | 23 (0.9%) | 2370 (93.6%) |  |
| P-values are from analysis of variance (age, frailty index score), Kruskal Wallis (Charlson Index), or chi-square (all others); NR: not reportable due to small cell size limitations (<6); *reported as mean (standard deviation); **reported as median (interquartile range) | | | | | |  |

**Appendix 7 – Incidence and Predictors of Postoperative Discharge Recovery State at Day 14**

At 14-days after surgery, 85.0% of patient were home, 12.8% were still hospitalized and 1.2% had died. Only 1.1% were in a rehabilitation center and <1% were in a long-term care facility (Table 2-main manuscript). Construct validity was supported by directionally expected associations between pre-specified predictors and greater odds of being in a worse Postoperative Discharge Recovery State (Figure). Greater preoperative frailty, comorbidity, and operative stress were strongly associated with stepwise increases in the odds of being in a worse Postoperative Discharge Recovery State at 14-days, while each decile increase in age was also significantly associated with being in a worse Postoperative Discharge Recovery State. Females were significantly more likely to be in a better Postoperative Discharge Recovery State than males, while patients having general surgical procedures were directionally more likely to be in a worse Postoperative Discharge Recovery State than all other surgical specialties, and significantly worse than thoracic, urology and vascular procedures.

**
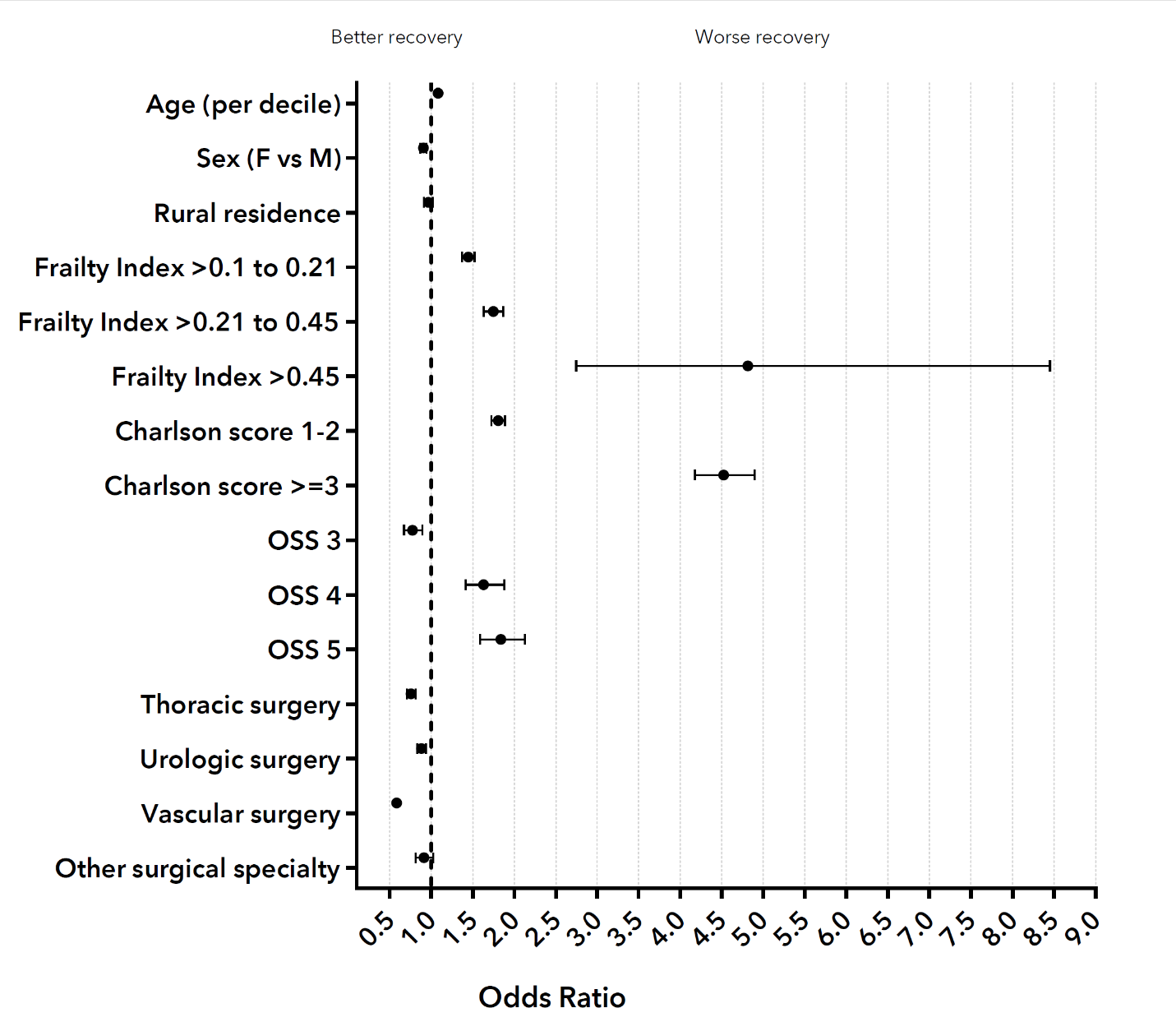
Figure – This figure presents the odds ratios and 95% confidence intervals from a multivariable ordinal logistic regression model containing baseline characteristics postulated to be associated with 14-day Postoperative Discharge Recovery State. The odds ratio can be interpreted as the relative odds of being in a worse Postoperative Discharge Recovery State per decile increase for age, or relative to the reference category. OSS: Operative Stress Score.**

**Appendix 8 – Predictive validity of Postoperative Discharge Recovery State at 90-days in predicting days alive and at home at 365 days**

The table below presents the results from a linear regression model with log-transformed days alive and at home in the year after surgery as the dependent variable and Postoperative Discharge Recovery Status at 90-days as a categorical independent variable. The ratio of means (RoM) represents the relative association of each 90-day category compared to being at home on the 90^th^ day after surgery; values less than 1 represent fewer days alive at home for a given category compared to the reference category. The R^2^ value for this model was 0.646, suggesting that Postoperative Discharge Recovery State at 90-days explains approximately 65% of the observed variation in days alive at home at 365 days.

| **90-day status** | **RoM** | **95%CI** |
| --- | --- | --- |
| Home | Ref | Ref |
| Rehabilitation | 0.24 | 0.23 to 0.25 |
| Long term care | 0.07 | 0.07 to 0.08 |
| Hospital | 0.32 | 0.31 to 0.33 |
| Dead | 0.01 | 0.01 to 0.01 |
| CI: confidence interval; Ref: Reference category; RoM: Ratio of Means | | |

**Appendix 9 – Smoothed calibration plots**

Smoother calibration plots represent the Loess-smoothed association between observed vs. predicted probability of residence in each discharge recovery state at the highlighted time point. A dashed line of ideal calibration moves from the bottom left to upper right. Smoothed calibration lines above the line of ideal calibration represent under-prediction of risk, whereas below the line of ideal calibration represents over-prediction of risk.


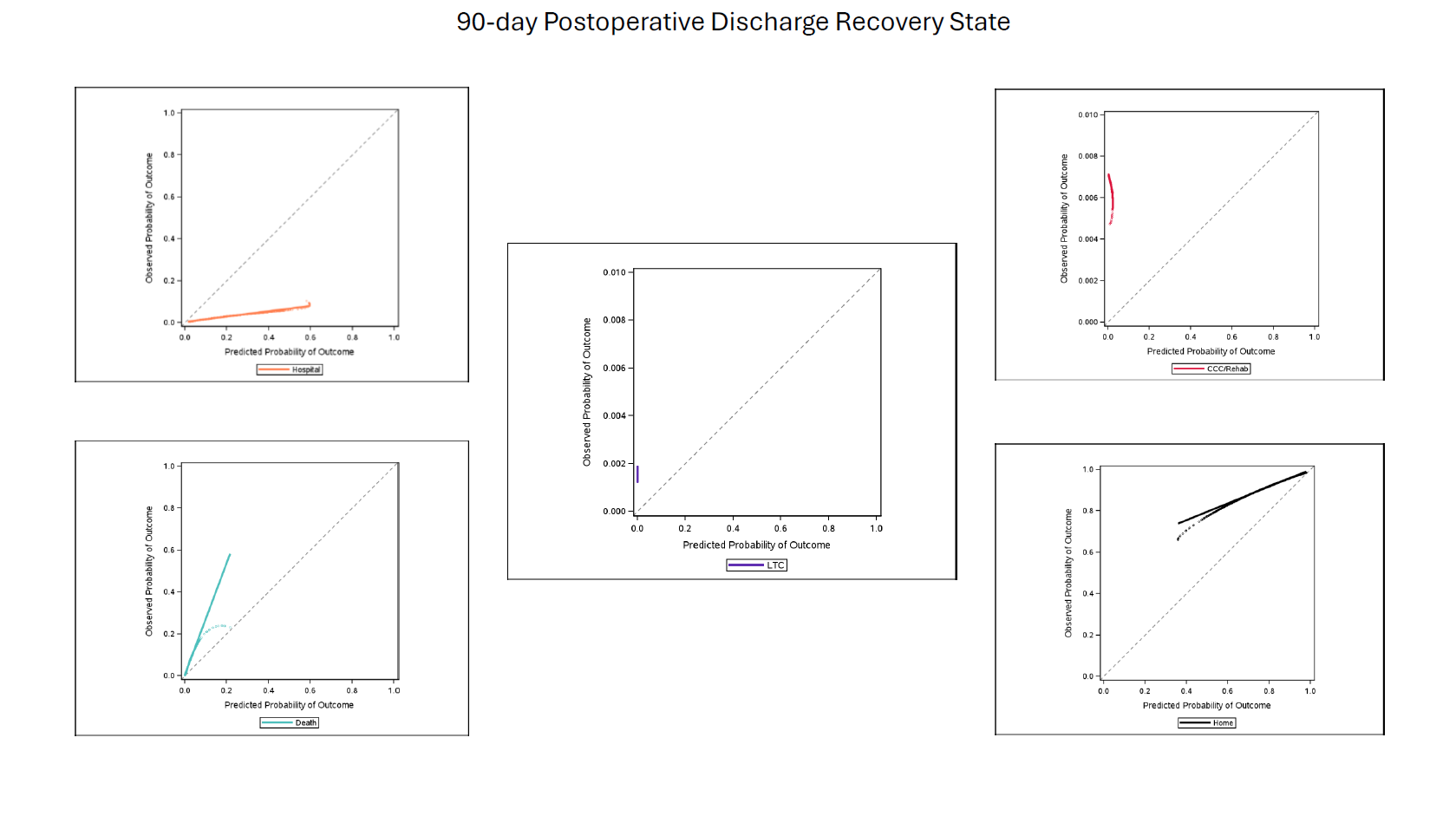


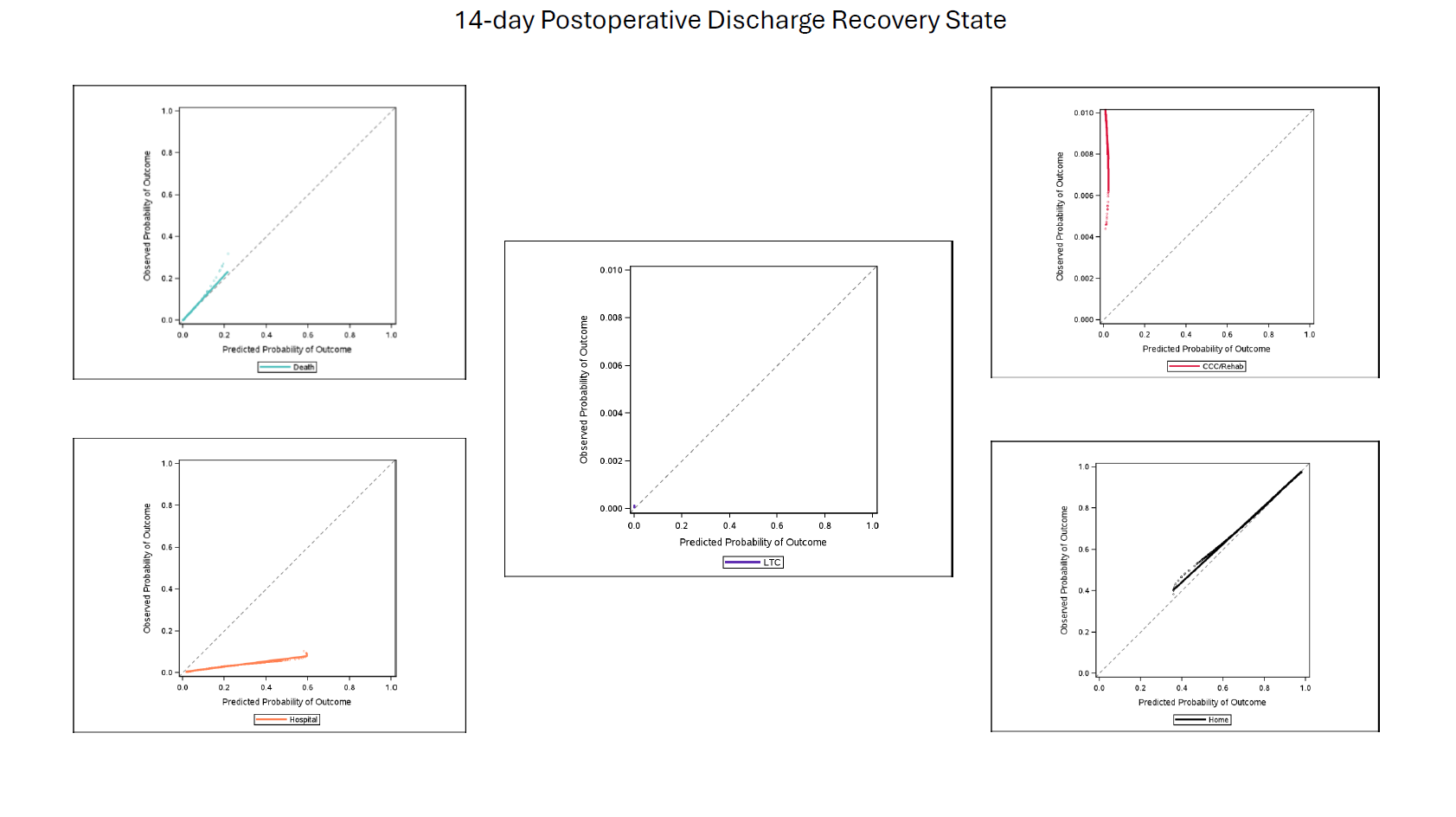


**Appendix 10 – Estimated sample sizes for trials with 1:1 allocation**

Calculations assume alpha=0.05 and power of 0.80 or 0.90. The binary outcome collapses dead, alive in hospital, long-term care and rehabilitation into a single category. The 4-category approach was done post-hoc by collapsing long-term care and rehabilitation into a single category.

| **OR** | **Power** | **Binary** | **Ordinal** | **Difference** |
| --- | --- | --- | --- | --- |
| *90-days: 5 categories* | | | | |
| **0.90** | 0.9 | 65698 | 65626 | 72 |
| **0.90** | 0.8 | 49075 | 49021 | 54 |
| **0.75** | 0.9 | 8812 | 8803 | 9 |
| **0.75** | 0.8 | 6582 | 6575 | 7 |
| **0.50** | 0.9 | 1518 | 1516 | 2 |
| **0.50** | 0.8 | 1134 | 1133 | 1 |
| *90-days: 4 categories** | | | | |
| **0.90** | 0.9 | 65698 | 65626 | 72 |
| **0.90** | 0.8 | 49075 | 49022 | 53 |
| **0.75** | 0.9 | 8812 | 8803 | 9 |
| **0.75** | 0.8 | 6583 | 6575 | 8 |
| **0.50** | 0.9 | 1518 | 1516 | 2 |
| **0.50** | 0.8 | 1134 | 1133 | 1 |
| *14 days: 5 categories* | | | | |
| **0.90** | 0.9 | 29679 | 29579 | 100 |
| **0.90** | 0.8 | 22170 | 22095 | 75 |
| **0.75** | 0.9 | 3981 | 3967 | 14 |
| **0.75** | 0.8 | 2974 | 2964 | 10 |
| **0.50** | 0.9 | 686 | 683 | 3 |
| **0.50** | 0.8 | 512 | 511 | 1 |
