# Supplementary material for: Description and validation of the Postoperative Discharge Recovery State outcome: a patient-partnered population-based cohort study
Source: Br J Anaesth. 2025 Jun 25;135(3):746–54. doi: 10.1016/j.bja.2025.05.020 (PMC12489357; doi:10.1016/j.bja.2025.05.020)
Supplement: Multimedia component 2 [file mmc2.docx]

**Description and validation of the Postoperative Discharge Recovery State outcome: a patient-partnered population-based cohort study**

Emily Hladkowicz, Gurlavine Kidd, Alana Flexman, Allan Garland, Julie Hallet, Daniel Kobewka, Matthew McGarr, Robert Talarico, Carl van Walraven, Duminda N. Wijeysundera, Camilla L. Wong, Daniel I McIsaac, on behalf of the NOTIFY Investigators* and the Canadian Perioperative Anesthesia Clinical Trials Group

* Listed in Appendix

**Corresponding Author: Daniel I McIsaac MD, MPH, FRCPC**
Professor of Anesthesiology and Pain Medicine, University of Ottawa
Scientist, Ottawa Hospital Research Institute
Email: [dmcisaac@toh.ca](mailto:dmcisaac@toh.ca)
Phone-613-761-4940/Fax-613-761-5209
Department of Anesthesiology, The Ottawa Hospital Civic Campus
Room B311, 1053 Carling Ave
Ottawa, ON K1Y 4E9

**Funding**: This work was supported by The Canadian Institutes of Health Research (FRN-190194) and The Ottawa Hospital Academic Medical Organization (TOH-24-015).

**Abstract**

**Background:** Older adults prioritize independent return home after surgery. Most discharge outcomes are binary composites that do not incorporate temporal information. We defined and validated a novel ordinal outcome, the Postoperative Discharge Recovery State, and prioritize its temporal measurement, to overcome these limitations.

**Methods:** This retrospective cohort study was conducted with patient partnership. Adults >65 yr having major, elective, non-cardiac, non-orthopaedic surgery were identified from 2012 to 2022 using linked, routinely collected data in Ontario, Canada. Construct, convergent and predictive validity were estimated. A multivariable ordinal regression model was derived and internally-externally validated.

**Results:** We included 84,422 older adult surgical patients. At the patient-prioritized postoperative day 90, the distribution of patients across Postoperative Discharge Recovery State categories was: 1) dead (2,718; 3.2%); 2) hospitalized (1,696; 2.0%); 3) in long-term care (179; 0.2%); 4) in rehabilitation (593; 0.7%); and 5) at home (79,236; 93.9%). Directionally expected associations with baseline characteristics supported construct validity. Consistency in associations over time supported reliability. Relationships with days alive and at home supported convergent (ρ=0.373) and predictive (fewer days at home with worse recovery state) validity. A prespecified ordinal logistic regression model had inadequate accuracy (c-statistic 0.700, poor calibration) to support its clinical use.

**Conclusion:** The Postoperative Discharge Recovery State is a 5-level ordinal outcome that can be applied at key time points after surgery to quantify the proportion of patients in patient-prioritized discharge locations. Validity and reliability support utility, but further development will be required to maximize information gain relative to binary outcomes.

**Keywords:** geriatrics, surgery, epidemiology, validation

**Editor’s key points:**

- Older patients need guidance about the likelihood of returning to independent living at home after planned surgery. Current scales are often binary (home/not a home) and do not capture data of a timeframe that is meaningful to patients.
- The Postoperative Discharge Recovery State outcome is a 5-level ordinal variable what was developed from population data with the help of patient partners. The worst category is death, followed by being hospitalized, in a long-term care facility, in a rehabilitation centre or home.
- A total 84,422 older adult surgical patients from Ontario, Canada, were included. Ninety days after surgery, 93.9% of patients were at home. The outcome exhibited construct validity, reliability, convergent and predictive validity. However it had inadequate accuracy to support its clinical use.
- Further development will be required to maximize information gain relative to binary outcomes.

**Introduction**

Failing to return to independent living at home after major surgery is a primary concern for older patients (i.e., those aged >65 yr).^1–3^ However, many older surgical patients report losing a meaningful degree of function after surgery,^4^ 1 in 5 report development of a new disability,^5^ and more than 20% may not achieve their goal of returning home after major surgery.^6,7^ Unfortunately, existing postoperative outcome measures fail to capture variations in post-discharge states that are meaningful to older adults.

Given the risk of loss of independence faced by older surgical patients, many experience uncertainty when deciding whether to have surgery.^8^ A key source of uncertainty expressed by individuals with lived experience is a lack of consistent and accurate communication of expected recovery timelines and the likelihood of postoperative return to independence. This uncertainty is reflected in the literature, where available studies typically define loss of independence or non-home discharge using ‘catch-all’ composites, where in-patient rehabilitation, respite care, long-term care admission and even death are treated equivalently as a single, dichotomous outcome.^9–14^ Additionally, these dichotomous composite outcome definitions are typically applied only at hospital discharge, meaning a home discharge 4 days after surgery is treated equivalently to a home discharge 4 months after surgery.

As use of routinely collected healthcare data continues to expand in perioperative health services research, patient-centred outcomes such as days alive and at home (DAH) are recommended by core outcome sets^15^ and can be feasibly captured from routinely collected data.^16,17^ Such outcomes can help to provide patients with more meaningful and information rich insights. However, the DAH outcome receives relatively lower priority ratings from older adults compared to other commonly studied perioperative outcomes.^1^ This may be due, at least in part, to the DAH outcome being variably defined in perioperative studies, with some authors reporting days alive and out of hospital, and others reporting a true count of days alive and at home by accounting for days in rehabilitation or other non-hospital institutional locations as non-home days.^18^ As such, there can be confusion expressed by patients regarding where they would be if not at home. Given the opportunities that routinely collected healthcare data present to inform prognosis, health system design, and efficient conduct of registry linked clinical trials, the objectives of this study were to partner with a group of older adults who had lived experience of having surgery: (1) develop a meaningful outcome capturing temporal and recovery state priorities of older individuals using routinely collected data (Postoperative Discharge Recovery State), (2) describe population-level distributions of this outcome; and (3) provide initial estimates of validity^19^ for the Postoperative Discharge Recovery State outcome in older individuals having non-cardiac, non-orthopaedic, major, elective surgeries.

**Methods**

*Design and setting*

This was a population-based retrospective cohort study using linked health administrative data in Ontario, Canada. A protocol was prespecified and registered.^20^ Data were accessed through ICES, an independent research institute that houses routinely collected health data for Ontario, where residents universally receive publicly funded insurance for physician and hospital services, as well as post-acute and long-term care. All data were linked deterministically across datasets listed in Appendix 1 using an anonymized, unique identifier over a 10-year period from 2012 to 2022 (which represented the most recent date with complete data). Reporting follows standards for observational studies using routinely collected data.^21,22^ Ethical review of analyses of anonymized and routinely collected are legally waived under section 45 of Ontario health privacy legislation.

*Patient and partner engagement*

Patient and partner engagement is reported using appropriate checklists.^23^ Our team used an established integrated knowledge translation approach to partner with patients and knowledge users to jointly design the protocol, execute the study and disseminate findings.^24,25^ Specifically, we partnered with a group of six older adults with lived experience of having surgery. The lead patient partner (GK) is an established member of the research program who partnered in this study and facilitated the snowball sampling technique to engage five additional patient partners. We developed a questionnaire (Appendix 2) that was first piloted with GK who provided important feedback that informed the final version of the tool. A member of the research team (EH) connected with each patient partner to explain the purpose of the questionnaire and how their responses would be interpreted and used to support the research study. To inform the creation of an outcome variable that could be captured in health administrative data and meaningfully inform surgical decision making for older individuals, we met with patient partners individually and administered the questionnaire to understand their perspectives on: 1) the specific types of information that they felt were lacking when faced with a decision about having surgery, 2) the specific considerations they had regarding location and timing of recovery after surgery; 3) how they wished to receive information about location and time of recovery in the clinical setting; and 4) to review and discuss how their information needs aligned with available metrics in routinely collected health data (specific details are provided in Appendix 2). Open-ended questions were analyzed using content analysis and the study team met to review and interpret the findings. Key insights identified by patient partners that informed development of the outcome were: 1) that 90 days after surgery represented a key time point in postoperative recovery (i.e., partners stated that they were willing to endure up to 3 months of difficult recovery and/or time away from home, whereas more than 3 months would likely be unacceptable); 2) the importance of knowing their likely physical location each week over the first 90 days after surgery, which would help them to plan important aspects of their personal lives (such as arranging at home supports, taking time off of work, coordinating support for other responsibilities (e.g., home maintenance, pet care); and 3) that consensus could be reached on a clear order of preference in terms of discharge locations that could be identified in routinely collected health data (as described in Appendix 2), which supported the face and content validity of the outcome.

*Cohort*

We identified all Ontario residents, aged 65 yr or older who presented for planned, major non-cardiac, non-orthopaedic surgery using a set of validated and well-studied, representative, intermediate to high stress procedures (vascular: open aortic aneurysm repair, endovascular aortic aneurysm repair, peripheral artery bypass, carotid endarterectomy; general: large bowel resection, pancreaticoduodenectomy, liver resection; thoracic: lung resection, oesophageal resection; urology: nephrectomy, cystectomy).^16,26–29^ Procedural severity was classified using the Operative Stress Score (OSS^30^; see Appendix 3 for all codes and categorizations). Elective surgery status was ensured by limiting inclusion to hospitalizations with an elective admission status and a qualifying surgery performed within 1 day of admission (to exclude surgeries that were the result of complications during an initially non-surgical elective admission). For patients with multiple elective surgeries during the study period, one randomly selected surgery was selected, allowing us to create a patient-level dataset. Individuals living in a long-term care facility before surgery were excluded (as they are uncommon in elective surgery populations and would be expected to return to their long-term care facility).^31^

*Outcome*

Based on patient partner engagement we defined a novel outcome, the Postoperative Discharge Recovery State, which we primarily defined at 90 days after surgery. The Postoperative Discharge Recovery State is a 5-level ordinal variable where the worst category is death, followed by being hospitalized, in a long-term care facility, in a rehabilitation centre, or home (specific definitions Appendix 4). As secondary outcomes, we captured the outcome on the last day of each week in the first 12 weeks after surgery. In the case that a state transition (e.g., rehabilitation to home) occurred on an outcome ascertainment date, we attributed the day to the worse outcome state. To support estimation of convergent and predictive validity, we also collected DAH values.

*Covariates*

We collected covariates that our team prioritized as being relevant to team members’ (i.e., patient partners, clinical partners and scientific collaborators) perspectives, that would be available to clinicians in the preoperative phase of care to support prognosis and decision making, and that were postulated to be associated with the Postoperative Discharge Recovery State outcome. These variables included age (in years), sex (male vs. female), rural vs. urban residence, frailty index score (based on the validated preoperative frailty index^32^), comorbidities (Charlson Comorbidity Index^33^), OSS,^30^ and surgical specialty (general, thoracic, vascular, urology, other; from the most responsible provider field in the hospital record).

*Analysis*

Descriptive statistics were compiled for each covariate by the specific Postoperative Discharge Recovery State at 90 days and compared using analysis of variance (normal continuous variables), Kruskall-Wallis tests (continuous skewed variables), or chi-squared tests (categorical variables). All analyses were performed using SAS v9.4 for Windows (SAS Institute, Cary NC). An alpha of 0.05 was used to suggest strong statistical evidence of a difference; as analyses were exploratory, no multiplicity adjustments were applied.^34^ Sample size considerations are described in Appendix 5.

We estimated the proportion of individuals (along with 95% confidence intervals [CI]^35^) in each Postoperative Discharge Recovery State at 90 days after surgery, as well as at the end of each week in the 12 weeks following surgery to describe the distribution of the ordinal Postoperative Discharge Recovery State across the 3 months following surgery.

Construct validity was evaluated by estimating the associations (using generalized odds ratios [OR]), where values > 1 suggest higher odds of being in a worse Postoperative Discharge Recovery State) between pre-specified predictor variables (listed in the *Covariates* section) and a patient’s Postoperative Discharge Recovery State at 90 days (primary analysis) and 14 days (secondary analysis) using multivariable ordinal logistic regression models. By performing analyses at 90- and 14-days, we aimed to evaluate whether associations were temporally consistent (which would support reliability), with 14 days identified as a time where most elective surgical patients following an uncomplicated source would be expected to achieve discharge. Convergent validity was estimated in comparison to DAH at 90 days using Spearman correlation coefficients. Predictive validity was estimated using linear regression with log-transformed DAH at 365-days as the dependent variable and Postoperative Discharge Recovery State at 90 days as the sole, categorical predictor.

We estimated the predictive accuracy of our set of prespecified baseline covariates in predicting the expected Postoperative Discharge Recovery State for each patient using ordinal logistic regression. Measures of predictive accuracy included the ordinal c-statistic (a measure of discrimination) and explained variance using the Nagelkeke R^2^ value at 90- and 14-days after surgery. Optimism and potential out of sample performance were estimated using an internal-external validation approach where the model was derived in the first temporal half of data (2012-2016) and validated in the second half (2015-2022).

*Estimation of sample size and power calculations for future studies*

To demonstrate how use of the Postoperative Discharge Recovery State outcome could inform future study design, we used the R (R Foundation for Statistical Computing, Vienna, Austria) package ‘posamsize’ to estimate the required sample size for trials designed to detect ORs of 0.9, 0.75 and 0.5 with 80% and 90% power, using a fixed alpha of 5% and 1:1 allocation between comparator groups.

**Results**

Characteristics of patient partners who contributed to defining the Postoperative Discharge Recovery State are provided in Appendix 2.

We identified 84,422 older patients having an elective, major inpatient non-cardiac, non-orthopaedic surgery between April 1^st^, 2012 and March 31^st^, 2022. Mean cohort age was 74.8 (SD 6.3) years; general surgical procedures were most common, and most procedures were an OSS or 4 or 5, representing significant expected surgical stress. Individuals who were at home 90 days after surgery were younger, more likely to be female, and lived with lower frailty scores (Table 1; OSS and surgical specialty row percentages are provided in Appendix 6.

*Incidence and Predictors of Postoperative Discharge Recovery State*

At 90 days after surgery, 93.9% of older individuals were alive and at home, with 3.2% having died, 2.0% still being in hospital and less than 1% being in a rehabilitation or long-term care centre (Table 2). Construct validity was supported by directionally expected associations between pre-specified predictors and higher odds of being in a worse Postoperative Discharge Recovery State (Figure 1). Higher levels of preoperative frailty, age, and comorbidity were strongly associated with stepwise increases in the odds of being in a worse Postoperative Discharge Recovery State at 90 days. Higher operative stress was associated with being in a worse Postoperative Discharge Recovery State, but the effect estimate for OSS 5 was slightly lower than for OSS 4. The association of surgical specialty and Postoperative Discharge Recovery State was inconsistent. Females were significantly more likely to be in a better Postoperative Discharge Recovery State than males. Data for 14 days after surgery are provided in Appendix 7.
*Convergent and predictive validity*

We estimated moderate correlation between the Postoperative Discharge Recovery State at 90 days and the count of DAH at 90 days (Spearman correlation coefficient 0.373, *P*<0.001). The Postoperative Discharge Recovery State at 90 days was associated with fewer DAH in the 365-days after surgery, with those who died having the lowest relative mean DAH (ratio of means (RoM) 0.011, 95% CI 0.010 to 0.011) compared to those who were at home by postoperative day 90 (Appendix 8).

*Predictive accuracy of prespecified multivariable models*

At 90 days, discrimination of the multivariable ordinal regression model was moderate (validation ordinal c-statistic 0.708), while postulated variables together explained 7.5% of observed variance (Nagelkerke R^2^ of 0.075). Calibration was poor for each possible state (Appendix 8).

At 14-days, discrimination of the multivariable ordinal regression model was marginally lower (validation ordinal c-statistic 0.695), while postulated variables explained a higher degree of variance (Nagelkerke R^2^ of 0.100). Calibration was improved for death and home states relative to 90-day predictions (Appendix 9).

*Postoperative Discharge Recovery State over time*

Figure 2 provides the distribution of Postoperative Discharge Recovery States over each of the 12 weeks after surgery. Large weekly decreases in hospitalized patients stabilized at 6 to 7 weeks after surgery, while the proportion who had died similarly increased weekly until week 6 to 7. The proportion in rehabilitation increased in week 2 and appeared to remain stable thereafter.

*Sample size and power considerations for future studies*

Estimated sample sizes for studies with 1:1 allocation between comparator groups ranged from 65,626 for an anticipated OR=0.90 with 90% power to 1,133 for an anticipated OR=0.50 with 80% power (Table 3). However, under no set of assumptions did use of the ordinal Postoperative Discharge Recovery State reduce the required sample size by more than 100 participants compared to a dichotomization of home vs. not home at 90 days (Appendix 10).

**Discussion**

In this population-based cohort study, conducted with direct patient partnership and guided by integrated knowledge translation, we developed, described, and evaluated the validity of the Postoperative Discharge Recovery State outcome. Results supported the construct, convergent, and predictive validity of the Postoperative Discharge Recovery State. For patients and clinicians, our data suggest that 85% of older patients were alive and at home by 14 days after surgery, and 94% were home by postoperative day 90. As a clinical trial outcome, this ordinal outcome does not appear to provide meaningful efficiency over binary outcomes. Parsimonious predictive modelling suggests that more complex modelling approaches may be required for accurate patient-level prognostication. Patients should continue to be engaged to develop, evaluate and select informative and meaningful outcomes in health services research.

Successfully returning home after surgery is a top priority for older adults.^1^ Unfortunately, current approaches do not provide accurate or meaningful prognostic information in many cases. This gap is largely attributable to two factors, whereby most measures meant to reflect recovery of independence simply dichotomize discharge disposition at the end of the index hospitalization. This approach means that an individual discharged home on postoperative day 2, who subsequently died on postoperative day 10 would be classified as having a better discharge outcome than someone who was discharged to a rehabilitation centre on postoperative day 7 and returned home independently on postoperative day 21. In contrast, the Postoperative Discharge Recovery State utilizes the ordered nature of possible discharge locations and applies this ordinal approach at patient-prioritized time points to provide a more informative ascertainment of postoperative recovery. Using this approach in population-based data, our results suggest that rates of adverse discharge reported using routinely collected data likely over-estimate the risk of patient-relevant non-home discharge, as almost 94% of older adults in our study were alive and at home at 90 days, which our partners identified as a key time point for patient-centred decision making.

Novel outcomes must demonstrate multiple aspects of validity and reliability. Working directly with older people with lived experience having surgery supports the face and content validity of the Postoperative Discharge Recovery State to reflect the informational needs expressed by patients using naturally ordered and validated data. Construct validity, which assesses whether a measure captures its intended metric was supported by consistent and clinically expected associations between baseline characteristics and being in a worse Postoperative Discharge Recovery State. Moderate correlation between the Postoperative Discharge Recovery State and a count of DAH at 90 days suggests that these two measures capture related, but distinct aspects of postoperative recovery. Being in a worse Postoperative Discharge Recovery State at 90 days was associated with experiencing fewer DAH in the year after surgery, supporting predictive validity. Therefore, both the Postoperative Discharge Recovery State and DAH could provide complimentary information in perioperative research and decision making. Lastly, the Postoperative Discharge Recovery State appears to be reliable over time, as associations between baseline characteristics and Postoperative Discharge Recovery State were similar in direction and magnitude at 14 and 90 days after surgery.

While our data support the validity and reliability of the Postoperative Discharge Recovery State, further analyses also highlight limitations. Having an accurate model to estimate personalized expected risk of being in each different Postoperative Discharge Recovery State at 90 days could help to support higher quality preoperative decision making. However, our pre-specified parsimonious ordinal regression model did not have adequate accuracy to support clinical uptake. Future efforts need to consider less parsimonious predictor sets and more complex regression modelling techniques.^36^ The uneven distribution of participants across the categories of the Postoperative Discharge Recovery States likely explains the minimal gains in efficiency estimated in exemplar sample size estimates. These results also highlight the importance of patient-reported measures reflecting independence, such as the World Health Organization Disability Assessment Schedule (WHODAS),^37^ which is a core outcome that is highly prioritized by patients and would provide more granularity among patients already at home who often struggle to maintain independence. Efforts to routinely capture such outcomes could support meaningful and patient-centered health system optimization.

*Limitations*

The Postoperative Discharge Recovery State was defined and evaluated in routinely collected data from one Canadian province, which may limit generalizability, especially to jurisdictions with different data infrastructures, or where rehabilitation and long-term care services are not publicly funded. In fact, some Canadians may also pay privately for rehabilitation services, which could result in misclassification. Further validation in other surgical populations (e.g., cardiac, orthopaedic, emergency) is required. Convergent and predictive validity using patient-reported measures would increase confidence in the outcome’s validity, as would external validation of results and prognostic models. While all partners agreed on the ordering of outcome categories, some partners expressed that possible states of disability that we could not measure could be worse than death. Sparse numbers in rehabilitation and long-term care categories across time may require collapsing these categories in future analyses, but further patient engagement is required to inform such decisions.

**Conclusion**

The Postoperative Discharge Recovery State is a patient-centred 5-level ordinal outcome variable that can be captured in routinely collected data at various patient-prioritized time points after surgery. While this study supports the outcome’s validity and reliability, further development is required to provide an information rich, routinely collected outcome reflecting postoperative recovery.

**References**

1. Abdellatif S, Hladkowicz E, Lalu MM, Boet S, Gagne S, McIsaac DI. Patient-prioritization of routine and patient-reported postoperative outcome measures: a prospective, nested cross-sectional study. *Can J Anaesth*. 2021;ahead of p. doi:10.1007/s12630-022-02191-7

2. Fried TR, Bradley EH, Towle VR, Allore H. Understanding the treatment preferences of seriously ill patients. *N Engl J Med*. 2002;346(14):1061-1066. doi:10.1056/NEJMsa012528

3. Hladkowicz E, Auais M, Kidd G, McIsaac DI, Miller J. “I can’t imagine having to do it on your own”: a qualitative study on postoperative transitions in care from the perspectives of older adults with frailty. *BMC Geriatr*. 2023;23(1):848. doi:10.1186/s12877-023-04576-9

4. Berian JR, Mohanty S, Ko CY, Rosenthal RA, Robinson TN. Association of Loss of Independence With Readmission and Death After Discharge in Older Patients After Surgical Procedures. *JAMA Surg*. 2016;151(9):e161689. doi:10.1001/jamasurg.2016.1689

5. McIsaac DI, Taljaard M, Bryson GL, et al. Frailty as a Predictor of Death or New Disability After Surgery: A Prospective Cohort Study. *Ann Surg*. 2020;271(2):283-289. doi:10.1097/SLA.0000000000002967

6. McIsaac DI, Beaulé PE, Bryson GL, Van Walraven C. The impact of frailty on outcomes and healthcare resource usage after total joint arthroplasty: a population-based cohort study. *Bone Jt J*. 2016;98-B(6):799-805. doi:10.1302/0301-620X.98B6.37124

7. Watt J, Tricco AC, Talbot-Hamon C, et al. Identifying older adults at risk of harm following elective surgery: a systematic review and meta-analysis. *BMC Med*. 2018;16(1):2. doi:10.1186/s12916-017-0986-2

8. Oresanya LB, Lyons WL, Finlayson E. Preoperative assessment of the older patient: A narrative review. *JAMA - J Am Med Assoc*. 2014;311(20):2110-2120. doi:10.1001/jama.2014.4573

9. Hyder JA, Wakeam E, Habermann EB, Hess EP, Cima RR, Nguyen LL. Derivation and validation of a simple calculator to predict home discharge after surgery. *J Am Coll Surg*. 2014;218(2):226-236. doi:10.1016/j.jamcollsurg.2013.11.002

10. AlHilli MM, Schold JD, Kelley J, Tang AS, Michener CM. Preoperative assessment using the five-factor modified frailty index: A call for standardized preoperative assessment and prehabilitation services in gynecologic oncology. *Gynecol Oncol*. 2022;166(3):379-388. doi:10.1016/j.ygyno.2022.07.003

11. Mohanty S, Rosenthal RA, Russell MM, Neuman MD, Ko CY, Esnaola NF. Optimal Perioperative Management of the Geriatric Patient: A Best Practices Guideline from the American College of Surgeons NSQIP and the American Geriatrics Society. *J Am Coll Surg*. 2016;222(5):930-947. doi:10.1016/j.jamcollsurg.2015.12.026

12. Oldmeadow LB, McBurney H, Robertson VJ. Predicting risk of extended inpatient rehabilitation after hip or knee arthroplasty. *J Arthroplasty*. 2003;18(6):775-779. doi:10.1016/s0883-5403(03)00151-7

13. Hammer M, Althoff FC, Platzbecker K, et al. Discharge Prediction for Patients Undergoing Inpatient Surgery: Development and validation of the DEPENDENSE score. *Acta Anaesthesiol Scand*. 2021;65(5):607-617. doi:10.1111/aas.13778

14. Pathak P, Sahara K, Spolverato G, Pawlik TM. Development and validation of risk stratification tool for prediction of increased dependence using preoperative frailty after hepatopancreatic surgery. *Surgery*. 2022;172(2):683-690. doi:10.1016/j.surg.2022.03.021

15. Moonesinghe SR, Jackson AIR, Boney O, et al. Systematic review and consensus definitions for the Standardised Endpoints in Perioperative Medicine initiative: patient-centred outcomes. *Br J Anaesth*. 2019;123(5):664-670. doi:10.1016/j.bja.2019.07.020

16. Jerath A, Austin PC, Wijeysundera DN. Days Alive and Out of Hospital: Validation of a Patient-centered Outcome for Perioperative Medicine. *Anesthesiology*. 2019;131(1):84-93. doi:10.1097/ALN.0000000000002701

17. McIsaac DI, Talarico R, Jerath A, Wijeysundera DN. Days alive and at home after hip fracture: a cross-sectional validation of a patient-centred outcome measure using routinely collected data. *BMJ Qual Saf*. Published online July 30, 2021:bmjqs-2021-013150. doi:10.1136/bmjqs-2021-013150

18. Ribeiro T, Malhotra AK, Bondzi-Simpson A, et al. Days at home after surgery as a perioperative outcome: scoping review and recommendations for use in health services research. *Br J Surg*. 2024;111(12):znae278. doi:10.1093/bjs/znae278

19. Mokkink LB, Terwee CB, Patrick DL, et al. The COSMIN checklist for assessing the methodological quality of studies on measurement properties of health status measurement instruments: an international Delphi study. *Qual Life Res*. 2010;19(4):539-549. doi:10.1007/s11136-010-9606-8

20. McIsaac DI. Protocol for a population-based cohort study to describe and validate the ordinal Postoperative Discharge Recovery State outcome. Published online June 6, 2024. doi:10.17605/OSF.IO/RPWJ5

21. Benchimol EI, Smeeth L, Guttmann A, et al. The REporting of studies Conducted using Observational Routinely-collected health Data (RECORD) Statement. *PLOS Med*. 2015;12(10):e1001885. doi:10.1371/journal.pmed.1001885

22. von Elm E, Altman DG, Egger M, Pocock SJ, Gøtzsche PC, Vandenbroucke JP. Strengthening the Reporting of Observational Studies in Epidemiology (STROBE) statement: guidelines for reporting observational studies. *BMJ*. 2007;335(7624):806-808. doi:10.1136/bmj.39335.541782.AD

23. Staniszewska S, Brett J, Simera I, et al. GRIPP2 reporting checklists: tools to improve reporting of patient and public involvement in research. *BMJ*. Published online August 2, 2017:j3453. doi:10.1136/bmj.j3453

24. Boland L, Kothari A, McCutcheon C, Graham ID. Building an integrated knowledge translation (IKT) evidence base: colloquium proceedings and research direction. *Health Res Policy Syst*. 2020;18(1):8. doi:10.1186/s12961-019-0521-3

25. Canadian Institutes of Health Research. Guide to Knowledge Translation Planning at CIHR: Integrated and End-of-Grant Approaches. Accessed June 8, 2018. http://www.cihr-irsc.gc.ca/e/45321.html#a3

26. McIsaac DI, Bryson GL, van Walraven C. Association of Frailty and 1-Year Postoperative Mortality Following Major Elective Noncardiac Surgery: A Population-Based Cohort Study. *JAMA Surg*. 2016;151(6):538-545. doi:10.1001/jamasurg.2015.5085

27. Wijeysundera DN, Beattie WS, Austin PC, Hux JE, Laupacis A. Non-invasive cardiac stress testing before elective major non-cardiac surgery: population based cohort study. *BMJ*. 2010;340(jan28 3):b5526-b5526. doi:10.1136/bmj.b5526

28. McIsaac DI, Huang A, Wong CA, Wijeysundera DN, Bryson GL, van Walraven C. Effect of Preoperative Geriatric Evaluation on Outcomes After Elective Surgery: A Population-Based Study. *J Am Geriatr Soc*. 2017;65(12):2665-2672. doi:10.1111/jgs.15100

29. Jerath A, Austin PC, Ko DT, et al. Socioeconomic Status and Days Alive and Out of Hospital after Major Elective Noncardiac Surgery. *Anesthesiology*. 2020;132(4):713-722. doi:10.1097/ALN.0000000000003123

30. Shinall MC, Arya S, Youk A, et al. Association of Preoperative Patient Frailty and Operative Stress with Postoperative Mortality. *JAMA Surg*. 2020;155(1):1-9. doi:10.1001/jamasurg.2019.4620

31. McIsaac DI, Wijeysundera DN, Huang A, Bryson GL, van Walraven C. Association of the Hospital Volume of Frail Surgical Patients Cared for with Outcomes after Elective, Major Noncardiac Surgery: A Retrospective Population-based Cohort Study. *Anesthesiology*. 2017;126(4):602-613. doi:mcisac bmj qual

32. McIsaac DI, Wong CA, Huang A, Moloo H, van Walraven C. Derivation and Validation of a Generalizable Preoperative Frailty Index Using Population-based Health Administrative Data. *Ann Surg*. 2019;270(1):102-108. doi:10.1097/SLA.0000000000002769

33. Quan H, Sundararajan V, Halfon P, et al. Coding algorithms for defining comorbidities in ICD-9-CM and ICD-10 administrative data. *Med Care*. 2005;43(11):1130-1139.

34. Althouse AD. Adjust for Multiple Comparisons? It’s Not That Simple. *Ann Thorac Surg*. 2016;101(5):1644-1645. doi:10.1016/j.athoracsur.2015.11.024

35. Agresti A, Coull BA. Approximate is Better than “Exact” for Interval Estimation of Binomial Proportions. *Am Stat*. 1998;52(2):119-126. doi:10.1080/00031305.1998.10480550

36. Edlinger M, van Smeden M, Alber HF, Wanitschek M, Van Calster B. Risk prediction models for discrete ordinal outcomes: calibration and the impact of the proportional odds assumption. 2021;(April):1-27. doi:10.1002/sim.9281

37. Shulman MA, Myles PS, Chan MTV, McIlroy DR, Wallace S, Ponsford J. Measurement of Disability-free Survival after Surgery. *Anesthesiology*. 2015;122(3):524-536. doi:10.1097/ALN.0000000000000586

**Appendix (Editor: at the end of the paper rather than in the supplement**

*NOTIFY Investigators*

Soha Abdellatif, MD, Jamal Alkadri, MD, MSc, , Sylvie Aucoin, MD, MSc, Rebecca Auer, MD, MSc, Chantal Backman, PhD, MHA, BScN, Scott Beattie, MD, PhD, Weiwei Beckerleg, MD, MPH, Sylvain Boet, MD, PhD, Rodney H. Breau, MD, MSc, Gregory L. Bryson, MD, MSc, Francois Carrier, MD, MSc, Tyler Chesney, MD, MSc, Renee El-Gabalawy, PhD, MA, Alan Forster, MD, MSc, Sylvain Gagne, MD, Alexa Grudzinski, MD, Jayna Holroyd-Leduc, MD, Allen Huang, MDCM, Shirey Huang, MD, MSc, Angela Jerath, MD, MSc, Janny Ke, MD, MSc, Rachel Khadaroo, MD, PhD, Karim Ladha, MD, MSc, Manoj Lalu, MD, PhD, Luke T. Lavallée, MD, MSc, Susan Lee, MD, Grace Ma, MD, MSc, Guillaume Martel, MD, MSc, Kelly Mayson, MD, Arnaud Mbadjeu Hondjeu, MD, Sarah McIsaac, MBBCh, MEd, Husein Moloo, MD, MSc, MPH, Barbara Power, MD, Krista Reich, MD, MSc, Derek Roberts, MD, PhD, Jason Sutherland, PhD, Monica Taljaard, PhD, Peter Tanuseputro, MD, MHSc, CCFP

**Author contributions:**

| EH | Conceptualized, interpreted, drafted and final approval |
| --- | --- |
| GK | Conceptualized, interpreted, drafted and final approval |
| AF | Conceptualized, interpreted, drafted and final approval |
| AG | Conceptualized, interpreted, drafted and final approval |
| JH | Conceptualized, interpreted, drafted and final approval |
| DK | Conceptualized, interpreted, drafted and final approval |
| MM | Conceptualized, interpreted, analyzed, drafted and final approval |
| CVW | Conceptualized, interpreted, drafted and final approval |
| RT | Conceptualized, interpreted, analyzed, drafted and final approval |
| DNW | Conceptualized, interpreted, drafted and final approval |
| DIM | Conceptualized, interpreted, analyzed, drafted and final approval |
| CLW | Conceptualized, interpreted, drafted and final approval |
| SA | Conceptualized, interpreted, revised, final approval |
| JA | Conceptualized, interpreted, revised, final approval |
| SA | Conceptualized, interpreted, revised, final approval |
| RA | Conceptualized, interpreted, revised, final approval |
| CB | Conceptualized, interpreted, revised, final approval |
| SB | Conceptualized, interpreted, revised, final approval |
| WB | Conceptualized, interpreted, revised, final approval |
| SB | Conceptualized, interpreted, revised, final approval |
| RHB | Conceptualized, interpreted, revised, final approval |
| GLB | Conceptualized, interpreted, revised, final approval |
| FC | Conceptualized, interpreted, revised, final approval |
| TC | Conceptualized, interpreted, revised, final approval |
| RE | Conceptualized, interpreted, revised, final approval |
| AF | Conceptualized, interpreted, revised, final approval |
| SG | Conceptualized, interpreted, revised, final approval |
| AG | Conceptualized, interpreted, revised, final approval |
| JH | Conceptualized, interpreted, revised, final approval |
| AH | Conceptualized, interpreted, revised, final approval |
| SH | Conceptualized, interpreted, revised, final approval |
| AJ | Conceptualized, interpreted, revised, final approval |
| JK | Conceptualized, interpreted, revised, final approval |
| RK | Conceptualized, interpreted, revised, final approval |
| KL | Conceptualized, interpreted, revised, final approval |
| ML | Conceptualized, interpreted, revised, final approval |
| LTL | Conceptualized, interpreted, revised, final approval |
| SL | Conceptualized, interpreted, revised, final approval |
| GM | Conceptualized, interpreted, revised, final approval |
| GM | Conceptualized, interpreted, revised, final approval |
| KM | Conceptualized, interpreted, revised, final approval |
| AMH | Conceptualized, interpreted, revised, final approval |
| SM | Conceptualized, interpreted, revised, final approval |
| HM | Conceptualized, interpreted, revised, final approval |
| BP | Conceptualized, interpreted, revised, final approval |
| KR | Conceptualized, interpreted, revised, final approval |
| DR | Conceptualized, interpreted, revised, final approval |
| JS | Conceptualized, interpreted, revised, final approval |
| MT | Conceptualized, interpreted, revised, final approval |
| PT | Conceptualized, interpreted, revised, final approval |

**Table 1 – Demographics stratified by 90-day Post-operative Discharge Recovery State**

|  | **Dead** | **Hospitalized** | **Long term care** | **Rehabilitation** | **Alive at Home** | ***P*-value** |
| --- | --- | --- | --- | --- | --- | --- |
|  | n=2,718 | n=1,696 | n=179 | n=593 | n=79,236 |  |
| **Age*** | 77.3 (6.9) | 75.2 (6.3) | 80.8 (6.6) | 77.6 (6.6) | 74.6 (6.2) | <0.001 |
| **Female sex** | 973 (35.8%) | 640 (37.7%) | 105 (58.7%) | 235 (39.6%) | 32,248 (40.7%) | <0.001 |
| **Rural residence** | 410 (3.2%) | 272 (2.1%) | 31 (0.2%) | 56 (0.4%) | 12,221 (94.1%) | 0.002 |
| **Frailty index score*†** | 0.19 (0.08) | 0.18 (0.08) | 0.22 (0.10) | 0.20 (0.08) | 0.14 (0.07) | <0.001 |
| **Charlson Index**** | 1 (1-2) | 1 (0-2) | 1 (1-2) | 1 (1-2) | 1 (0-1) | <0.001 |
| **Operative Stress Score** |  |  |  |  |  | <0.001 |
| *2* | 42 (1.5%) | 30 (1.8%) | 7 (3.9%) | 16 (2.7%) | 2,199 (2.8%) |  |
| *3* | 773 (28.4%) | 522 (30.8%) | 66 (36.9%) | 203 (34.2%) | 32,119 (40.5%) |  |
| *4* | 1,498 (55.1%) | 878 (51.8%) | 91 (50.8%) | 287 (48.4%) | 33,625 (42.4%) |  |
| *5* | 405 (14.9%) | 266 (15.7%) | 15 (8.4%) | 87 (14.7%) | 11,293 (14.3%) |  |
| **Surgical Specialty** |  |  |  |  |  | <0.001 |
| *General* | 1,486 (54.7%) | 850 (50.1%) | 107 (59.8%) | 268 (45.2%) | 40,033 (50.5%) |  |
| *Vascular* | 493 (18.1%) | 348 (20.5%) | 48 (26.8%) | 173 (29.2%) | 18,000 (22.7%) |  |
| *Thoracic* | 280 (10.3%) | 206 (12.1%) | NR | NR | 7,819 (9.9%) |  |
| *Urology* | 379 (13.9%) | 241 (14.2%) | 13 (7.3%) | 83 (14.0%) | 11,014 (13.9%) |  |
| *Other* | 80 (2.9%) | 51 (3.0%) | 8 (4.5%) | 23 (3.9%) | 2,370 (3.0%) |  |
| P-values are from analysis of variance (age, frailty index score), Kruskal Wallis (Charlson Index), or chi-square (all others); NR: not reportable due to small cell size limitations (<6); *reported as mean (standard deviation); **reported as median (interquartile range); **†**frailty index ranges 0 to 1. | | | | | |  |

**Table 2 –Postoperative Discharge Recovery State at 14 and 90 days after surgery**

| **Postoperative Discharge Recovery State** | **Postoperative Day 14** | **Postoperative Day 90** |
| --- | --- | --- |
| ***Dead*** | 1.2% (1.2 to 1.2%) | 3.2% (3.1 to 3.3%) |
| ***Hospitalized*** | 12.8% (12.5 to 13.0%) | 2.0% (1.9 to 2.1%) |
| ***Long term care*** | 0.02% (0.02 to 0.04%) | 0.2% (0.2 to 0.3%) |
| ***Rehabilitation*** | 1.1% (1.0 to 1.1%) | 0.7% (0.7 to 0.8%) |
| ***Alive at Home*** | 85.0% (84.8 to 85.2%) | 93.9% (93.7 to 94.0%) |

**Data presented as proportion and 95% confidence interval**

**Table 3 – Estimated sample sizes required to detect small (odds ratio (OR) 0.9), moderate (OR 0.75), and large (OR 0.5) effect sizes with a power of 0.8 or 0.9.**

| Odds ratio | Power | Sample size for binary home vs not | Sample size for ordinal PDRS | Difference in participants required |
| --- | --- | --- | --- | --- |
| 0.9 | 0.9 | 65,698 | 65,626 | 72 |
| 0.9 | 0.8 | 49,075 | 49,021 | 54 |
| 0.75 | 0.9 | 8,812 | 8,803 | 9 |
| 0.75 | 0.8 | 6,582 | 6,575 | 7 |
| 0.5 | 0.9 | 1,518 | 1,516 | 2 |
| 0.5 | 0.8 | 1,134 | 1,133 | 1 |
| All calculations assume 1:1 allocation and 5% alpha. PDRS: Postoperative Discharge Recovery State | | | | |

**FIGURE LEGENDS**

Figure 1 – This figure presents the odds ratios and 95% confidence intervals from a multivariable ordinal logistic regression model containing baseline characteristics postulated to be associated with 90-day. The odds ratio can be interpreted as the relative odds of being in a worse Postoperative Discharge Recovery State per decile increase for age, or relative to the reference category. OSS: Operative Stress Score.

Figure 2 - This figure shows the proportion of patients in each Postoperative Discharge Recovery State on the 7^th^ day of each of the 12 weeks after surgery.
